# Relaxed Boundary Conditions in Poisson–Nernst–Planck Models: Identifying Critical Potentials for Multiple Cations

**DOI:** 10.3390/membranes15110339

**Published:** 2025-11-13

**Authors:** Xiangshuo Liu, Henri Ndaya, An Nguyen, Zhenshu Wen, Mingji Zhang

**Affiliations:** 1 College of Mathematics and Systems Science, Shandong University of Science and Technology, Qingdao 266510, China; liuxiangshuo@sdust.edu.cn; 2 Department of Mathematics, New Mexico Institute of Mining and Technology, Socorro, NM 87801, USA; henri.ndaya@student.nmt.edu (H.N.); an.nguyen.1@student.nmt.edu (A.N.); 3 School of Mathematical Sciences, Huaqiao University, Quanzhou 362021, China

**Keywords:** PNP, ionic flows, critical potentials, 34A26, 34B16, 34D15, 37D10, 92C35

## Abstract

Ion channels are protein pores that regulate ionic flow across cell membranes, enabling vital processes such as nerve signaling. They often conduct multiple ionic species simultaneously, leading to complex nonlinear transport phenomena. Because experimental techniques provide only indirect measurements of ion channel currents, mathematical models—particularly Poisson–Nernst–Planck (PNP) equations—are indispensable for analyzing the underlying transport mechanisms. In this work, we examine ionic transport through a one-dimensional steady-state PNP model of a narrow membrane channel containing multiple cation species of different valences. The model incorporates a small fixed charge distribution along the channel and imposes relaxed electroneutrality boundary conditions, allowing for a slight charge imbalance in the baths. Using singular perturbation analysis, we first derive approximate solutions that capture the boundary-layer structure at the channel—reservoir interfaces. We then perform a regular perturbation expansion around the neutral reference state (zero fixed charge with electroneutral boundary conditions) to obtain explicit formulas for the steady-state ion fluxes in terms of the system parameters. Through this analytical approach, we identify several critical applied potential values—denoted $V_{k}^{a}$ (for each cation species *k*), $V^{b}$, and $V^{c}$—that delineate distinct transport regimes. These critical potentials govern the sign of the fixed charge’s influence on each ion’s flux: depending on whether the applied voltage lies below or above these thresholds, a small positive permanent charge will either enhance or reduce the flux of each ion species. Our findings thus characterize how a nominal fixed charge can nonlinearly modulate multi-ion currents. This insight deepens the theoretical understanding of nonlinear ion transport in channels and may inform the interpretation of current–voltage relations, rectification effects, and selective ionic conduction in multi-ion channel experiments.

## 1. Introduction

Ion channels are large proteins embedded in cell membranes that control the flow of ions, which is vital for cellular communication, signal transduction, and coordinated cellular tasks [1,2,3,4]. Ion channel research focuses on two interlinked areas: structural characterization and ionic flow analysis. The geometry of a channel and the distribution of its permanent and polarization charges define its physical configuration. Many ion channels feature a cylindrical domain with a non-uniform cross-section. Within a large class of these channels, amino acid side chains are primarily located in a “short” and “narrow” region [5,6,7,8]. The structure of ion channels allows them to selectively and rapidly facilitate the passive diffusion of specific ions across cell membranes.

The permeation and selectivity of an ion channel are primarily defined by experimentally measured current-voltage (I-V) relations under different ionic conditions [2,9]. Experimental measurement of ionic flow is limited to input-output data [2], precluding direct observation of a channel’s internal dynamics. Therefore, mathematical modeling, particularly using Poisson–Nernst–Planck (PNP) systems, is crucial for understanding ion channel behavior. Recent advances in the mathematical analysis of the PNP system have provided valuable insights into this behavior [10,11,12,13,14,15,16,17,18,19,20,21,22].

### 1.1. Poisson–Nernst–Planck Model for Ionic Flows

A basic continuum model for ionic flows is the PNP system, which accounts for the medium’s structural properties by representing the aqueous environment as a dielectric continuum (see [23,24,25,26,27,28,29,30] and the reference therein). Under various conditions, the PNP system can be rigorously derived as a reduced model from approaches such as molecular dynamics [31], Boltzmann equations [32], and variational principles [33,34,35]. The PNP system is an appropriate model for both the analysis and numerical simulations of ionic flows, given the biological system’s key features.

In this work, we adopt the one-dimensional steady-state PNP model analyzed in [5] first proposed by [36], which reads, k=1,2,…,n, Please check the whole manuscript.

(1) $$\begin{array}{c} \begin{array}{cc} \; & \frac{1}{A(X)}\frac{d}{dX}\left(\varepsilon_{r}\left(X\right)\varepsilon_{0}A\left(X\right)\frac{d\Phi }{dX}\right)=-e\left(\sum_{s=1}^{n}z_{s}C_{s}+Q\left(X\right)\right),\\ \; & \frac{dJ_{k}}{dX}=0,\;-J_{k}=\frac{1}{k_{B}T}D_{k}\left(X\right)A\left(X\right)C_{k}\frac{d\mu_{k}}{dX},\;k=1,2,\ldots ,n, \end{array} \end{array}$$

where X∈[0,l] is the coordinate along the axis of the channel, A(X) is the area of cross-section of the channel over the location *X*, Q(X) is the permanent charge density of the channel, ϕ(X) is the electric potential, *e* is the elementary charge, for each k,$z_{k}$ is the valence, $C_{k}$ is the number density, $J_{k}$ is the flux density, $D_{k}\left(X\right)$ is the diffusion coefficient.

Equipped with system (1), we impose the following boundary conditions (see [11] for a reasoning), for k=1,2,…,n,

(2) $$\Phi \left(0\right)=V,\;\;C_{k}\left(0\right)=L_{k}>0;\;\Phi \left(l\right)=0,\;\;C_{k}\left(l\right)=R_{k}>0.$$

In our following discussion, we assume that the relative dielectric coefficient and the diffusion coefficient are constants, that is, $\varepsilon_{r}\left(X\right)=\varepsilon_{r}$ and $D_{i}\left(X\right)=D_{i}$. We also make a dimensionless rescaling following [8]. Let

(3) $$\begin{array}{c} \begin{array}{cc} \varepsilon^{2}= & \frac{\varepsilon_{r}\varepsilon_{0}k_{B}T}{e^{2}l^{2}C_{0}},\;x=\frac{X}{l},\;h\left(x\right)=\frac{A(X)}{l^{2}},\;D_{i}=lC_{0}D_{i};\\ \phi (x)= & \frac{e}{k_{B}T}\Phi \left(X\right),\;c_{i}\left(x\right)=\frac{C_{i}\left(X\right)}{C_{0}},\;J_{i}=\frac{J_{i}}{D_{i}};\\ V= & \frac{e}{k_{B}T}V,\;L_{i}=\frac{L_{i}}{C_{0}};\;R_{i}=\frac{R_{i}}{C_{0}}, \end{array} \end{array}$$

where $C_{0}$ is some characteristic number density.

The BVP (1) and (2) then becomes

(4) $$\begin{array}{c} \begin{array}{cc} \; & \frac{\varepsilon^{2}}{h(x)}\frac{d}{dx}\left(h\left(x\right)\frac{d}{dx}\phi \right)=-\left(z_{1}c_{1}+z_{2}c_{2}+z_{3}c_{3}+Q\left(x\right)\right),\\ \; & \frac{dc_{k}}{dx}+z_{k}c_{k}\frac{d\phi }{dx}=-\frac{J_{k}}{h(x)},\;\frac{dJ_{k}}{dx}=0,\;k=1,2,3 \end{array} \end{array}$$

with the boundary conditions, for i=1,2,3,

(5) $$\phi \left(0\right)=V,\;\;c_{i}\left(0\right)=L_{i}>0;\;\;\phi \left(1\right)=0,\;\;c_{i}\left(1\right)=R_{i}>0.$$

To end this section, we demonstrate that in ionic transport, flux reversal refers to a situation where the net flow of an ion species changes direction as conditions (such as the applied voltage) vary. In a simple system with one ion type, this occurs at the ion’s reversal potential (given by the Nernst equation), where electrical and diffusive forces exactly balance so that net flux is zero. For voltages below that critical value, the ion might flow in one direction; above it, the flow reverses direction. In multi-ion systems (like biological channels that conduct several ion species), multiple such critical voltages can arise, each delineating a change in transport regime. Our study in current work is naturally motivated by this observation, particularly, the identification of the critical voltages.

### 1.2. Electroneutrality Boundary Conditions vs. Boundary Layers

Accurate characterization of ion channel and biomimetic transistor function requires modeling the systems’ interactions with macroscopic ionic reservoirs [37,38,39,40]. The necessary macroscopic boundary conditions create boundary layers with distinct concentration and charge distributions. When these boundary layers extend into the device’s atomistically controlled region, they can significantly influence device behavior. Specifically, charge boundary layers can cause long-distance artifacts through their far-reaching electric fields [11].

While boundary layer effects are critical for accurately studying ion channel systems, many investigations of qualitative ionic flow properties simplify the problem by imposing electroneutrality boundary conditions at channel ends (see, e.g., [5,6,7,17,18,41,42,43,44,45,46,47,48,49,50,51,52,53,54,55,56,57,58,59]), given by

(6) $$\begin{array}{c} \sum_{k=1}^{n}z_{k}L_{k}=\sum_{k=1}^{n}z_{k}R_{k}=0. \end{array}$$

Condition (6), significantly reduces the complexity of ionic flow analysis. However, this simplification obscures the effects of boundary layers, which contain more detailed information about ionic behavior.

To understand ionic flow through membrane channels, boundary layer effects must be included in the analysis. Because electric potentials are sensitive to these boundary layers, a challenging but more realistic approach is to examine nearly neutral states. Following this framework, the author in [60] studied the qualitative properties of ionic flows using the cPNP system for one cation and one anion, with no permanent charges. For this, we introduce parameters σ and ρ with (σ,ρ)→(1,1) as follows:

(7) $$\begin{array}{c} \begin{array}{cc} \; & \begin{array}{c} z_{1}L_{1}+\ldots +z_{j}L_{j}=\sigma \left(-z_{j+1}L_{j+1}+\ldots +z_{n}L_{n}\right) \end{array},\\ \; & \begin{array}{c} z_{1}R_{1}+\ldots +z_{j}R_{j}=\rho \left(-z_{j+1}R_{j+1}-\ldots -z_{n}R_{n}\right), \end{array} \end{array} \end{array}$$

where $z_{s}>0$ for 1≤s≤j and $z_{s}<0$ for j+1≤s≤n. Note that σ=1=ρ in (7) implies the neutral state. Richer behavior of ionic flows was observed under this new setup. Later, the authors in [20,61,62,63] further studied the PNP system under different setups.

Building on established research that demonstrates the importance of the boundary layer for ionic flow properties, this work analyzes the boundary layer’s influence on the individual fluxes. This analysis is conducted under the specific condition of small, nonzero permanent charges, and relaxed boundary conditions as described by (7) for j=2 and n=3.

### 1.3. A Brief Summary of Our Work

Our work highlights how a multi-cation PNP channel with relaxed charge neutrality exhibits piecewise transport regimes controlled by critical voltages. At these critical potentials ($V_{k}^{a}$ for each ion, and $V^{b}$, $V^{c}$ from ion-ion coupling), the system undergoes qualitative changes: ion fluxes reverse direction or change their sensitivity to the channel’s charge. This explains why a fixed charge does not simply have a uniformly positive or negative effect, but instead switches from beneficial to detrimental influence at identifiable voltages. Such insights deepen our understanding of ion channel behavior—for instance, predicting inflection points or reversal potentials in I–V curves—and guide experimental observations of current rectification and selectivity in channels with multiple ionic species.

## 2. Mathematical Methods

To characterize the qualitative properties of the individual fluxes $J_{k}\left(V;\sigma ,\rho \right)$, we employ a two-step perturbation approach. First, we treat the boundary value problem (4) and (5) as a singular perturbation problem. By analyzing its limiting fast and slow systems, we construct singular orbits to obtain approximate, explicit expressions for the fluxes (see [5]). Second, we perform a regular perturbation expansion on the resulting flux expressions around the point (σ,ρ)=(1,1).

### 2.1. Previous Results

We recall some results from [5], which are the starting point of our analysis. In [5], along $Q_{0}=0$, the authors obtained, for general boundary conditions,

$$\begin{array}{c} J_{k}\left(V;Q_{0}\right)=J_{k0}\left(V\right)+Q_{0}J_{k1}\left(V\right)+o\left(Q_{0}\right), \end{array}$$

with $J_{k0}\left(V\right)$ and $J_{k1}\left(V\right)$ given by, k=1,2,

(8) $$\begin{array}{c} \begin{array}{cc} \; & J_{k0}=\frac{C^{\left[0,r\right]}-C^{\left[3,l\right]}}{H\left(1\right)\left(\ln C^{\left[0,r\right]}-\ln C^{\left[3,l\right]}\right)}\frac{\ln C^{\left[0,r\right]}-\ln C^{\left[3,l\right]}-z\left(\phi^{\left[3,l\right]}-\phi^{\left[0,r\right]}\right)}{C^{\left[0,r\right]}-C^{\left[3,l\right]}e^{z\left(\phi^{\left[3,l\right]}-\phi^{\left[0,r\right]}\right)}}\\ & \;\times \left(c_{k}^{\left[0,r\right]}-c_{k}^{\left[3,l\right]}e^{z\left(\phi^{\left[3,l\right]}-\phi^{\left[0,r\right]}\right)}\right),\\ \; & J_{30}=-\frac{z}{z_{3}}\frac{C^{\left[0,r\right]}-C^{\left[3,l\right]}}{H\left(1\right)\left(\ln C^{\left[0,r\right]}-\ln C^{\left[3,l\right]}\right)}\left(\ln C^{\left[0,r\right]}-\ln C^{\left[3,l\right]}-z_{3}\left(\phi^{\left[3,l\right]}-\phi^{\left[0,r\right]}\right)\right),\\ \; & J_{k1}=\frac{c_{k}^{\left[0,r\right]}-c_{k}^{\left[3,l\right]}e^{z\left(\phi^{\left[3,l\right]}-\phi^{\left[0,r\right]}\right)}}{C^{\left[0,r\right]}-C^{\left[3,l\right]}e^{z\left(\phi^{\left[3,l\right]}-\phi^{\left[0,r\right]}\right)}}\frac{M\left(1+z\lambda \right)}{\left(z-z_{3}\right)H\left(1\right)}\left(z_{3}\left(1-N\right)\lambda +1\right),\\ \; & J_{31}=\frac{M\left(1+z_{3}\lambda \right)}{\left(z_{3}-z\right)H\left(1\right)}\left(z(1-N)\lambda +1\right). \end{array} \end{array}$$

Here, for j=1,2,3, with $L=L_{1}+L_{2}$, $R=R_{1}+R_{2}$, $C^{\left[j-1,r\right]}=c_{1}^{\left[j-1,r\right]}+c_{2}^{\left[j-1,r\right]},\;C^{\left[j,l\right]}=c_{1}^{\left[j,l\right]}+c_{2}^{\left[j,l\right]}$ and $C^{\left[k\right]}=c_{1}^{\left[k\right]}+c_{2}^{\left[k\right]},$

(9) $$\begin{array}{c} \begin{array}{cc} \phi^{\left[0,r\right]}= & V-\frac{1}{z-z_{3}}\ln\frac{-z_{3}L_{3}}{zL},\;c_{1}^{\left[0,r\right]}=L_{1}{\left(\frac{-z_{3}L_{3}}{zL}\right)}^{\frac{z}{z-z_{3}}},\;c_{2}^{\left[0,r\right]}=L_{2}{\left(\frac{-z_{3}L_{3}}{zL}\right)}^{\frac{z}{z-z_{3}}},\\ c_{3}^{\left[0,r\right]}= & L_{3}{\left(\frac{-z_{3}L_{3}}{zL}\right)}^{\frac{z_{3}}{z-z_{3}}},\;\phi^{\left[1,l\right]}=\phi^{\left[1\right]}-\frac{1}{z-z_{3}}\ln\frac{-z_{3}c_{3}^{\left[1\right]}}{zC^{\left[1\right]}},\;c_{1}^{\left[1,l\right]}=c_{1}^{\left[1\right]}{\left(\frac{-z_{3}c_{3}^{\left[1\right]}}{zC^{\left[1\right]}}\right)}^{\frac{z}{z-z_{3}}},\\ c_{2}^{\left[1,l\right]}= & c_{2}^{\left[1\right]}{\left(\frac{-z_{3}c_{3}^{\left[1\right]}}{zC^{\left[1\right]}}\right)}^{\frac{z}{z-z_{3}}},\;c_{3}^{\left[1,l\right]}=c_{3}^{\left[1\right]}{\left(\frac{-z_{3}c_{3}^{\left[1\right]}}{zC^{\left[1\right]}}\right)}^{\frac{z_{3}}{z-z_{3}}},\;\phi^{\left[2,r\right]}=\phi^{\left[2\right]}-\frac{1}{z-z_{3}}\ln\frac{-z_{3}c_{3}^{\left[2\right]}}{zC^{\left[2\right]}},\\ c_{1}^{\left[2,r\right]}= & c_{1}^{\left[2\right]}{\left(\frac{-z_{3}c_{3}^{\left[2\right]}}{zC^{\left[2\right]}}\right)}^{\frac{z}{z-z_{3}}},\;c_{2}^{\left[2,r\right]}=c_{2}^{\left[2\right]}{\left(\frac{-z_{3}c_{3}^{\left[2\right]}}{zC^{\left[2\right]}}\right)}^{\frac{z}{z-z_{3}}},\;c_{3}^{\left[2,r\right]}=c_{3}^{\left[2\right]}{\left(\frac{-z_{3}c_{3}^{\left[2\right]}}{zC^{\left[2\right]}}\right)}^{\frac{z_{3}}{z-z_{3}}},\\ \phi^{\left[3,l\right]}= & -\frac{1}{z-z_{3}}\ln\frac{-z_{3}R_{3}}{zR},\;c_{1}^{\left[3,l\right]}=R_{1}{\left(\frac{-z_{3}R_{3}}{zR}\right)}^{\frac{z}{z-z_{3}}},\;c_{2}^{\left[3,l\right]}=R_{2}{\left(\frac{-z_{3}R_{3}}{zR}\right)}^{\frac{z}{z-z_{3}}},\\ c_{3}^{\left[3,l\right]}= & R_{3}{\left(\frac{-z_{3}R_{3}}{zR}\right)}^{\frac{z_{3}}{z-z_{3}}},\;c_{1}^{\left[1,r\right]}=c_{1}^{\left[1\right]}e^{z(\phi^{\left[1\right]}-\phi^{\left[1,r\right]})},\;c_{2}^{\left[1,r\right]}=c_{2}^{\left[1\right]}e^{z(\phi^{\left[1\right]}-\phi^{\left[1,r\right]})},\\ c_{3}^{\left[1,r\right]}= & c_{3}^{\left[1\right]}e^{z_{3}\left(\phi^{\left[1\right]}-\phi^{\left[1,r\right]}\right)},\;c_{1}^{\left[2,l\right]}=c_{1}^{\left[2\right]}e^{z(\phi^{\left[2\right]}-\phi^{\left[2,l\right]})},\;c_{2}^{\left[2,l\right]}=c_{2}^{\left[2\right]}e^{z(\phi^{\left[2\right]}-\phi^{\left[2,l\right]})},\\ c_{3}^{\left[2,l\right]}= & c_{3}^{\left[2\right]}e^{z_{3}\left(\phi^{\left[2\right]}-\phi^{\left[2,l\right]}\right)},\;\lambda =\frac{\phi^{\left[0,r\right]}-\phi^{\left[3,l\right]}}{\ln C^{\left[0,r\right]}-\ln C^{\left[3,l\right]}},\;M=\frac{\left(C^{\left[3,l\right]}-C^{\left[0,r\right]}\right)\left(C_{0}^{\left[1\right]}-C_{0}^{\left[2\right]}\right)}{C_{0}^{\left[1\right]}C_{0}^{\left[2\right]}\left(\ln C^{\left[0,r\right]}-\ln C^{\left[3,l\right]}\right)},\\ N= & \frac{\ln C_{0}^{\left[2\right]}-\ln C_{0}^{\left[1\right]}}{M}=\frac{C_{0}^{\left[1\right]}C_{0}^{\left[2\right]}\left(\ln C^{\left[0,r\right]}-\ln C^{\left[3,l\right]}\right)\left(\ln C_{0}^{\left[2\right]}-\ln C_{0}^{\left[1\right]}\right)}{\left(C^{\left[3,l\right]}-C^{\left[0,r\right]}\right)\left(C_{0}^{\left[1\right]}-C_{0}^{\left[2\right]}\right)}. \end{array} \end{array}$$

Recall that $\left(\phi^{\left[1\right]},c_{1}^{\left[1\right]},c_{2}^{\left[1\right]},c_{3}^{\left[1\right]}\right)$ and $\left(\phi^{\left[2\right]},c_{1}^{\left[2\right]},c_{2}^{\left[2\right]},c_{3}^{\left[2\right]}\right)$ are the unknown values preassigned at $x=x_{1}$ and $x=x_{2}$ for $\left(\phi \left(x\right),c_{1}\left(x\right),c_{2}\left(x\right)c_{3}\left(x\right)\right)$.

### 2.2. Expressions of the Individual Fluxes as (σ,ρ)→(1,1)

For $\phi^{L},\;\phi^{R},\;c_{k}^{L}$ and $c_{k}^{R}$ in (9), under the condition (7), one has

$$\begin{array}{cc} \phi^{L}= & V-\frac{\ln\sigma }{z-z_{3}},\;c_{1}^{L}=L_{1}\sigma^{\frac{z}{z-z_{3}}},\;c_{2}^{L}=L_{2}\sigma^{\frac{z}{z-z_{3}}},\;c_{3}^{L}=L_{3}\sigma^{\frac{z_{3}}{z-z_{3}}},\\ c^{L}= & c_{1}^{L}+c_{2}^{L}=L\sigma^{\frac{z}{z-z_{3}}},\;\phi^{R}=-\frac{\ln\rho }{z-z_{3}},\;c_{1}^{R}=R_{1}\rho^{\frac{z}{z-z_{3}}},\;c_{2}^{R}=R_{2}\rho^{\frac{z}{z-z_{3}}},\\ c_{3}^{R}= & R_{3}\rho^{\frac{z_{3}}{z-z_{3}}},\;c^{R}=c_{1}^{R}+c_{2}^{R}=R\rho^{\frac{z}{z-z_{3}}}. \end{array}$$

Furthermore, as (σ,ρ)→(1,1), up to the first order with $t=\frac{L}{R}$ and $s=\frac{L_{3}}{R_{3}}$, one has, for k=1,2,

(10) $$\begin{array}{c} \begin{array}{cc} J_{k0}\left(V;\sigma ,\rho \right)= & \frac{D_{k}\left(L_{k}-R_{k}e^{-zV}\right)\left(\ln t+zV\right)}{H\left(1\right)\left(t-e^{-zV}\right)\ln^{2}t}\left(\left(t-1\right)\ln t+\frac{z}{z-z_{3}}f\left(t\right)\right),\\ J_{30}\left(V;\sigma ,\rho \right)= & \frac{D_{3}R_{3}\left(\ln s+z_{3}V\right)}{H\left(1\right)\ln^{2}s}\left(\left(s-1\right)\ln s+\frac{z_{3}}{z-z_{3}}f\left(s\right)\right),\\ J_{k1}\left(V;\sigma ,\rho \right)= & \frac{D_{k}\left(L_{k}-R_{k}e^{-zV}\right)\left(\ln t+zV\right)}{\left(z-z_{3}\right)RH\left(1\right)\left(t-e^{-zV}\right)}(J_{110}+J_{111}\left(\sigma -1\right)\\ & -J_{111}\left(\rho -1\right)),\\ J_{31}\left(V;\sigma ,\rho \right)= & -\frac{D_{3}\left(\ln s+z_{3}V\right)}{\left(z-z_{3}\right)H\left(1\right)}\left(J_{310}+J_{311}\left(\sigma -1\right)-J_{311}\left(\rho -1\right)\right). \end{array} \end{array}$$

Here

(11) $$\begin{array}{c} \begin{array}{cc} f(x)= & (x\ln x-x+1)(\sigma -1)+(x-1-\ln x)(\rho -1),\\ J_{110}= & -\frac{1}{\omega \left(\alpha \right)\omega \left(\beta \right)\ln^{3}t}(z_{3}(\omega \left(\alpha \right)\omega \left(\beta \right)\ln t\ln\frac{\omega (\beta )}{\omega (\alpha )}+\left(\beta -\alpha \right){\left(t-1\right)}^{2})V\\ & +\left(\beta -\alpha \right){\left(t-1\right)}^{2}\ln t),\\ J_{111}= & \frac{z_{3}}{\left(z-z_{3}\right)\omega^{2}\left(\alpha \right)\omega^{2}\left(\beta \right)\ln^{4}t}(zD(\beta )V+F(\beta )),\\ J_{310}= & -\frac{1}{\omega \left(\alpha \right)\omega \left(\beta \right)\ln^{3}s}(z(\omega \left(\alpha \right)\omega \left(\beta \right)\ln s\ln\frac{\omega (\beta )}{\omega (\alpha )}+\left(\beta -\alpha \right){\left(s-1\right)}^{2})V\\ & +\left(\beta -\alpha \right){\left(s-1\right)}^{2}\ln s),\\ J_{311}= & \frac{z}{\left(z-z_{3}\right)\omega^{2}\left(\alpha \right)\omega^{2}\left(\beta \right)\ln^{4}s}\left(z_{3}\bar{D}\left(\beta \right)V+\bar{F}\left(\beta \right)\right), \end{array} \end{array}$$

where

(12) $$\begin{array}{c} \begin{array}{cc} \omega (x)= & t+x(1-t),\\ D(\beta )= & 2\omega^{2}\left(\alpha \right)\omega^{2}\left(\beta \right)\ln t\ln\frac{\omega (\beta )}{\omega (\alpha )}+(\left(t\ln^{2}t+3{\left(t-1\right)}^{2}\right)\omega \left(\alpha \right)\omega \left(\beta \right)\\ & +t\left(t-1\right)\left(-2t+(\alpha +\beta )(t-1)\right)\ln t)\left(\beta -\alpha \right),\\ F(\beta )= & \ln t(\omega^{2}\left(\alpha \right)\omega^{2}\left(\beta \right)\ln t\ln\frac{\omega (\beta )}{\omega (\alpha )}+(\frac{z}{z_{3}}t\left(t-1\right)\left((\alpha +\beta )(t-1)-2t\right)\ln t\\ & +\frac{2z+z_{3}}{z_{3}}{\left(t-1\right)}^{2}\omega \left(\alpha \right)\omega \left(\beta \right))\left(\beta -\alpha \right)),\\ \bar{D}\left(\beta \right)= & 2\omega^{2}\left(\alpha \right)\omega^{2}\left(\beta \right)\ln s\ln\frac{\omega (\beta )}{\omega (\alpha )}+(\left(s\ln^{2}s+3{\left(s-1\right)}^{2}\right)\omega \left(\alpha \right)\omega \left(\beta \right)\\ & +s\left(s-1\right)\left(-2s+(\alpha +\beta )(s-1)\right)\ln s)\left(\beta -\alpha \right),\\ \bar{F}\left(\beta \right)= & \ln s(\omega^{2}\left(\alpha \right)\omega^{2}\left(\beta \right)\ln s\ln\frac{\omega (\beta )}{\omega (\alpha )}+(\frac{z_{3}}{z}s\left(s-1\right)\left((\alpha +\beta )(s-1)-2s\right)\ln s\\ & +\frac{z+2z_{3}}{z}{\left(s-1\right)}^{2}\omega \left(\alpha \right)\omega \left(\beta \right))\left(\beta -\alpha \right)). \end{array} \end{array}$$

## 3. Results

Our main concern in this work is the effect on the individual fluxes with the nonzero but small permanent charges from boundary layers. In particular, we are interested in identifying some critical potentials, which play crucial role in the characterization of ionic flow properties.

For this, we introduce three functions $J_{kd}=J_{kd}\left(V;\sigma ,\rho ;Q_{0}\right)$ with k=1,2,3 as follows:

(13) $$\begin{array}{c} \begin{array}{cc} J_{1d}= & J_{1}\left(V;\sigma ,\rho \right)-J_{1}\left(V;1,1\right)=J_{1d}^{0}\left(V;\sigma ,\rho \right)+Q_{0}J_{1d}^{1}\left(V;\sigma ,\rho \right)+o\left(Q_{0}\right),\\ J_{2d}= & J_{2}\left(V;\sigma ,\rho \right)-J_{2}\left(V;1,1\right)=J_{2d}^{0}\left(V;\sigma ,\rho \right)+Q_{0}J_{2d}^{1}\left(V;\sigma ,\rho \right)+o\left(Q_{0}\right),\\ J_{3d}= & J_{3}\left(V;\sigma ,\rho \right)-J_{3}\left(V;1,1\right)=J_{3d}^{0}\left(V;\sigma ,\rho \right)+Q_{0}J_{3d}^{1}\left(V;\sigma ,\rho \right)+o\left(Q_{0}\right). \end{array} \end{array}$$

Here

(14) $$\begin{array}{c} \begin{array}{cc} J_{1d}^{0}= & \frac{zD_{1}\left(L_{1}-R_{1}e^{-zV}\right)}{\left(z-z_{3}\right)H\left(1\right)\ln^{2}t}\frac{\ln t+zV}{t-e^{-zV}}\left((t\ln t-t+1)(\sigma -1)+(t-1-\ln t)(\rho -1)\right),\\ J_{1d}^{1}= & \frac{D_{1}\left(L_{1}-R_{1}e^{-zV}\right)}{\left(z-z_{3}\right)RH\left(1\right)}\frac{\ln t+zV}{t-e^{-zV}}J_{111}\left(\sigma -\rho \right),\\ J_{2d}^{0}= & \frac{zD_{2}\left(L_{2}-R_{2}e^{-zV}\right)}{\left(z-z_{3}\right)H\left(1\right)\ln^{2}t}\frac{\ln t+zV}{t-e^{-zV}}\left((t\ln t-t+1)(\sigma -1)+(t-1-\ln t)(\rho -1)\right),\\ J_{2d}^{1}= & \frac{D_{2}\left(L_{2}-R_{2}e^{-zV}\right)}{\left(z-z_{3}\right)RH\left(1\right)}\frac{\ln t+zV}{t-e^{-zV}}J_{111}\left(\sigma -\rho \right),\\ J_{3d}^{0}= & \frac{z_{3}D_{3}R_{3}\left(\ln s+z_{3}V\right)}{\left(z-z_{3}\right)H\left(1\right)\ln^{2}s}\left((s\ln s-s+1)(\sigma -1)+(s-1-\ln s)(\rho -1)\right),\\ J_{3d}^{1}= & -\frac{D_{3}\left(\ln s+z_{3}V\right)}{\left(z-z_{3}\right)H\left(1\right)}J_{311}\left(\sigma -\rho \right), \end{array} \end{array}$$

where $J_{111}$ and $J_{311}$ are given in (11).

We also define some critical potentials by

**Definition** **1.** *We define $V_{1}^{a},\;V_{2}^{a}$, $V_{3}^{a},\;V^{b}$ and $V^{c}$ by*

(15) $$\begin{array}{c} \begin{array}{cc} V_{1}^{a}= & -\frac{1}{z}\ln\frac{L_{1}}{R_{1}},\;V_{2}^{a}=-\frac{1}{z}\ln\frac{L_{2}}{R_{2}},\;V_{3}^{a}=-\frac{1}{z_{3}}\ln\frac{L_{3}}{R_{3}},\\ V^{b}= & -\frac{1}{z}\frac{F(\beta )}{D(\beta )},\;V^{c}=-\frac{1}{z_{3}}\frac{\bar{F}\left(\beta \right)}{\bar{D}\left(\beta \right)}. \end{array} \end{array}$$

In our following discussion, we assume σ>ρ, and for the case with σ<ρ, it can be discussed similarly. We now focus on the sign of the leading term $J_{k1}^{d}\left(V;\sigma ,\rho \right)$ that contains small permanent charge effects under relaxed electroneutrality boundary conditions.

**Theorem** **1.** *Suppose that (σ,ρ)→(1,1). Then,*

(i) *With D(β)<0, for k=1,2,*
  (i1) *If $V_{k}^{a}>V^{b}$, then, $J_{kd}^{1}\left(V;\sigma ,\rho \right)>0$ for $V\in \left(-\infty ,V^{b}\right)\cup \left(V_{k}^{a},\infty \right)$, and $J_{kd}^{1}\left(V;\sigma ,\rho \right)<0$ for $V^{b}<V<V_{k}^{a}$, that is, the small (positive) permanent charge enhances $J_{kd}$ for $V\in \left(-\infty ,V^{b}\right)\cup \left(V_{k}^{a},\infty \right)$, and reduces $J_{kd}$ for $V^{b}<V<V_{k}^{a}$.*
  (i2) *If $V_{k}^{a}=V^{b}$, then, $J_{kd}^{1}\left(V;\sigma ,\rho \right)>0$ for any V, that is, the small (positive) permanent charge always enhances $J_{kd}$.*
  (i3) *If $V_{k}^{a}<V^{b}$, then, $J_{kd}^{1}\left(V;\sigma ,\rho \right)>0$ for $V\in \left(-\infty ,V_{k}^{a}\right)\cup \left(V^{b},\infty \right)$, and $J_{kd}^{1}\left(V;\sigma ,\rho \right)<0$ for $V_{k}^{a}<V<V^{b}$, that is, the small (positive) permanent charge enhances $J_{kd}$ for $V\in \left(-\infty ,V_{k}^{a}\right)\cup \left(V^{b},\infty \right)$, and reduces $J_{kd}$ for $V_{k}^{a}<V<V^{b}$.*
(ii) *With D(β)=0, for k=1,2,*
  (ii1) *If, in addition, F(β)>0, then, $J_{kd}^{1}\left(V;\sigma ,\rho \right)>0$ (resp. $J_{kd}^{1}\left(V;\sigma ,\rho \right)<0$) if $V<V_{k}^{a}$ (resp. $V>V_{k}^{a}$), that is, small (positive) permanent charge enhances (resp. reduces) $J_{kd}$ if $V<V_{k}^{a}$ (resp. $V>V_{k}^{a}$).*
  (ii2) *If, in addition, F(β)<0, then, $J_{kd}^{1}\left(V;\sigma ,\rho \right)>0$ (resp. $J_{kd}^{1}\left(V;\sigma ,\rho \right)<0$) if $V>V_{k}^{a}$ (resp. $V<V_{k}^{a}$), that is, small (positive) permanent charge enhances (resp. reduces) $J_{kd}$ if $V>V_{k}^{a}$ (resp. $V<V_{k}^{a}$).*
(iii) *With D(β)>0, for k=1,2*
  (iii1) *If $V_{k}^{a}>V^{b}$, then, $J_{kd}^{1}\left(V;\sigma ,\rho \right)>0$ for $V^{b}<V<V_{k}^{a}$, and $J_{kd}^{1}\left(V;\sigma ,\rho \right)<0$ for $V\in \left(-\infty ,V^{b}\right)\cup \left(V_{k}^{a},\infty \right)$, that is, the small (positive) permanent charge enhances $J_{kd}$ for $V^{b}<V<V_{k}^{a}$, and reduces $J_{kd}$ for $V\in \left(-\infty ,V^{b}\right)\cup \left(V_{k}^{a},\infty \right)$.*
  (iii2) *If $V_{k}^{a}=V^{b}$, then, $J_{kd}^{1}\left(V;\sigma ,\rho \right)<0$ for any V, that is, the small (positive) permanent charge always reduces $J_{kd}$;*
  (iii3) *If $V_{k}^{a}<V^{b}$, then, $J_{kd}^{1}\left(V;\sigma ,\rho \right)>0$ for $V_{k}^{a}<V<V^{b}$, and $J_{kd}^{1}\left(V;\sigma ,\rho \right)<0$ for $V\in \left(-\infty ,V_{k}^{a}\right)\cup \left(V^{b},\infty \right)$, that is, the small (positive) permanent charge enhances $J_{kd}$ if $V_{k}^{a}<V<V^{b}$, and reduces $J_{kd}$ for $V\in \left(-\infty ,V_{k}^{a}\right)\cup \left(V^{b},\infty \right)$.*

**Proof.**  The statements follow directly from the expression of $J_{kd}^{1}\left(V;\sigma ,\rho \right)=J_{k1}\left(V;\sigma ,\rho \right)-J_{k1}\left(V;1,1\right)$. □

Similarly, for $J_{3d}^{1}\left(V;\sigma ,\rho \right)=J_{31}\left(V;\sigma ,\rho \right)-J_{31}\left(V;1,1\right)$, the following result can be established.

**Theorem** **2.** *Suppose that (σ,ρ)→(1,1). Then,*

(i) *With $\bar{D}\left(\beta \right)<0$, one has*
  (i1) *If $V_{3}^{a}>V^{c}$, then, $J_{3d}^{1}\left(V;\sigma ,\rho \right)>0$ for $V<V^{c}$ or $V>V_{3}^{a}$, and $J_{3d}^{1}\left(V;\sigma ,\rho \right)<0$ if $V^{c}<V<V_{3}^{a}$, that is, small (positive) permanent charge enhances $J_{3d}\left(V;\sigma ,\rho ;Q_{0}\right)$ if $V<V^{c}$ or $V>V_{3}^{a}$, and reduces $J_{3d}\left(V;\sigma ,\rho ;Q_{0}\right)$ if $V^{c}<V<V_{3}^{a}$;*
  (i2) *if $V_{3}^{a}=V^{c}$, then, $J_{3d}^{1}\left(V;\sigma ,\rho \right)>0$ for any V, that is, small (positive) permanent charge always enhances $J_{3d}\left(V;\sigma ,\rho ;Q_{0}\right)$;*
  (i3) *if $V_{3}^{a}<V^{c}$, then, $J_{3d}^{1}\left(V;\sigma ,\rho \right)>0$ if $V<V_{3}^{a}$ or $V>V^{c}$, and $J_{3d}^{1}\left(V;\sigma ,\rho \right)<0$ if $V_{3}^{a}<V<V^{c}$, that is, small (positive) permanent charge enhances $J_{3d}\left(V;\sigma ,\rho ;Q_{0}\right)$ if $V<V_{3}^{a}$ or $V>V^{c}$, and reduces $J_{3d}\left(V;\sigma ,\rho ;Q_{0}\right)$ if $V_{3}^{a}<V<V^{c}$.*
(ii) *With $\bar{D}\left(\beta \right)=0$, one has*
  (ii1) *if, in addition, $\bar{F}\left(\beta \right)>0$, then, $J_{3d}^{1}\left(V;\sigma ,\rho \right)<0$ (resp. $J_{3d}^{1}\left(V;\sigma ,\rho \right)>0$) if $V<V_{3}^{a}$ (resp. $V>V_{3}^{a}$), that is, small (positive) permanent charge reduces (resp. enhances) $J_{3d}\left(V;\sigma ,\rho ;Q_{0}\right)$ if $V<V_{3}^{a}$ (resp. $V>V_{3}^{a}$);*
  (ii2) *if, in addition, $\bar{F}\left(\beta \right)<0$, then one has, $J_{3d}^{1}\left(V;\sigma ,\rho \right)>0$ (resp. $J_{3d}^{1}\left(V;\sigma ,\rho \right)<0$) if $V<V_{3}^{a}$ (resp. $V>V_{3}^{a}$), that is, small (positive) permanent charge enhances (resp. reduces) $J_{3d}\left(V;\sigma ,\rho ;Q_{0}\right)$ if $V<V_{3}^{a}$ (resp. $V>V_{3}^{a}$).*
(iii) *With $\bar{D}\left(\beta \right)>0$, one has*
  (iii1) *if $V_{3}^{a}>V^{c}$, then, $J_{3d}^{1}\left(V;\sigma ,\rho \right)>0$ if $V^{c}<V<V_{3}^{a}$, and $J_{3d}^{1}\left(V;\sigma ,\rho \right)<0$ if $V<V^{c}$ or $V>V_{3}^{a}$, that is, small (positive) permanent charge enhances $J_{3d}\left(V;\sigma ,\rho ;Q_{0}\right)$ if $V^{c}<V<V_{3}^{a}$, and reduces $J_{3d}\left(V;\sigma ,\rho ;Q_{0}\right)$ if $V<V^{c}$ or $V>V_{3}^{a}$;*
  (iii2) *if $V_{3}^{a}=V^{c}$, then, $J_{3d}^{1}\left(V;\sigma ,\rho \right)<0$ for any V, that is, small (positive) permanent charge always reduces $J_{3d}\left(V;\sigma ,\rho ;Q_{0}\right)$;*
  (iii3) *if $V_{3}^{a}<V^{c}$, then, $J_{3d}^{1}\left(V;\sigma ,\rho \right)>0$ if $V_{3}^{a}<V<V^{c}$, and $J_{3d}^{1}\left(V;\sigma ,\rho \right)<0$ if $V<V_{3}^{a}$ or $V>V^{c}$, that is, small (positive) permanent charge enhances $J_{3d}\left(V;\sigma ,\rho ;Q_{0}\right)$ if $V_{3}^{a}<V<V^{c}$, and reduces $J_{3d}\left(V;\sigma ,\rho ;Q_{0}\right)$ if $V<V_{3}^{a}$ or $V>V^{c}$.*

## 4. Discussion

Our analysis establishes that the critical potentials $V_{k}^{a},V^{b},$ and $V^{c}$ fundamentally organize the behavior of ionic fluxes in the PNP model, effectively partitioning the applied voltage domain into subregions of qualitatively distinct flux responses. In each such region, the presence of a small permanent charge has a consistent effect−either an enhancement or a suppression−on the flux of a given ionic species, and this effect reverses upon crossing one of the critical potentials. In particular, for each cation species *k*, the potential $V_{k}^{a}$ emerges as a threshold delineating whether a small positive fixed charge will augment or diminish that ion’s flow. This $V_{k}^{a}$ is determined explicitly by the model parameters (see Equation (12)), depending on the valence $z_{k}$ and the ratio of boundary concentrations for ion *k*. Consequently, $V_{k}^{a}$ can be interpreted as an intrinsic characteristic of ion *k*’s electrochemical environment in the channel, and it can be readily estimated from measurable quantities (ionic valences and feed concentrations). The potential $V_{k}^{a}$ thus serves as a practically accessible indicator of when the fixed charge’s influence on species k’s flux will change sign, offering direct insight into the channel’s ionic selectivity and flow regime.

In the case of two critical potentials governing a single species’ flux, those potentials carve the voltage axis into three distinct regions with alternating behavior. For example, when $V_{k}^{a}$ and $V^{b}$ are both present and ordered (say $V^{b}<V_{k}^{a}$), our results show that a small positive permanent charge enhances the flux of ion *k* for applied voltages *V* in the outer intervals (below $V^{b}$ or above $V_{k}^{a}$) while reducing the flux for intermediate voltages $V^{b}<V<V_{k}^{a}$. If the ordering is reversed ($V_{k}^{a}<V^{b}$), the enhancement and suppression regions swap accordingly−the fixed charge then boosts ion k’s flux only in the intermediate range $V_{k}^{a}<V<V^{b}$, and diminishes it outside that range. Notably, in the degenerate scenario $V_{k}^{a}=V^{b}$, no such partitioning occurs; the two thresholds coincide, and the small permanent charge exerts a unidirectional effect (pure enhancement or pure suppression) over the entire voltage domain with no reversals. In summary, the values of $V_{k}^{a}$ and $V^{b}$ act as switching points that determine whether adding a fixed charge will help or hinder the ionic flux in each voltage regime. This piecewise behavior underscores the nuanced, non-monotonic dependence of ionic currents on fixed charge in multi-ion PNP systems.

Interestingly, the qualitative pattern of flux enhancement vs. suppression is sensitive to underlying parameter regimes, as evidenced by the role of the composite functions D(β) and F(β) in our analysis. In one regime (characterized by D(β)<0), the fixed charge’s influence conforms to the pattern described above: it enhances flux outside a certain intermediate voltage window and suppresses flux within that window. In the opposite regime (D(β)>0), our results predict an inversion of this pattern. There, the small permanent charge boosts the ionic flux only within a bounded interval of voltages and reduces it for voltages below and above that interval. In other words, when D(β)>0 the enhancement is confined to the middle regime between $V_{k}^{a}$ and $V^{b}$, rather than the extremes. The presence of these two possible patterns (and the special case D(β)=0, which yields a single threshold $V_{k}^{a}$ with a single switch in behavior) highlights how subtle differences in model parameters can qualitatively alter the effect of fixed charge. Nevertheless, in all cases the critical potentials themselves ($V_{k}^{a}$ and $V^{b}$) remain the pivotal markers of transition: the sign of the fixed-charge effect on flux flips precisely at $V=V^{b}$ or $V=V_{k}^{a}$ as the system moves from one region to another. This provides a clear theoretical demarcation of regimes, which is valuable for understanding and predicting ionic flow characteristics.

For the anion species included in our model, an analogous set of conclusions is reached. In addition to its own $V_{3}^{a}$, the third ion’s flux is influenced by a second threshold potential $V^{c}$ that arises from the system’s parameters (through $\bar{D}\left(\beta \right)$ and $\bar{F}\left(\beta \right)$ in Equation (12)). Just as with the two cations, the relative ordering of $V_{3}^{a}$ and $V^{c}$ partitions the voltage domain into regions where the fixed charge has opposite effects on the flux of the anion. For one set of conditions (for instance, $\bar{D}\left(\beta \right)<0$), the small permanent charge enhances the flux of the anion for voltages either below the lower critical potential ($V<V^{c}$) or above the higher critical potential ($V>V_{3}^{a}$), while suppressing the flux in between those values. Under a different parameter regime ($\bar{D}\left(\beta \right)>0$), this relationship inverts: the fixed charge’s positive influence is restricted to the intermediate range between $V_{3}^{a}$ and $V^{c}$, and it reduces the flux of the anion outside that range. These findings for the anion reinforce the general principle that each identified critical potential corresponds to a reversal point in the flux response. Regardless of the number of ionic species, the set of critical potentials $\left\{V_{k}^{a},V^{b},V^{c},\ldots \right\}$ serve as reliable guideposts that delineate where a small permanent charge transitions from aiding to impeding the ionic flow of each species (and vice versa). The consistency of this piecewise behavior across all cations in the model underscores the robustness of our analytical approach in capturing the interplay between multiple ion species under the given boundary conditions.

From a theoretical standpoint, the existence of well-defined critical potentials in this multi-ion PNP system is significant. It demonstrates that complex ionic interactions and nonlinear coupling in the Poisson–Nernst–Planck equations can nevertheless yield tractable analytical criteria ($V_{k}^{a},V^{b},V^{c}$) governing qualitative outcomes. These results provide a rigorous explanation for a non-intuitive phenomenon: a single small fixed charge can either increase or decrease an ionic current depending on the value of the driving electrical potential. Without the framework developed in this work, one might have assumed a fixed charge has a uniform effect (either beneficial or detrimental) on ion transport. Instead, our analysis reveals a richer picture in which the sign of the effect is voltage-dependent and flips sharply at computable threshold values. This insight advances the qualitative understanding of ionic flow by pinpointing the conditions for which a passive channel modification (like introducing a tiny permanent charge) leads to improved conduction versus when it leads to suppression. Moreover, deriving explicit formulas for these threshold potentials (e.g., $V_{k}^{a}=-\frac{1}{z_{k}}\ln\left(L_{k}/R_{k}\right)$ for each ion *k*) not only confirms their dependence on fundamental parameters (such as valences and concentration ratios) but also lends mathematical clarity to the mechanisms of ion competition and selectivity in the model. The PNP system studied here, even under the relaxed electroneutrality boundary conditions, thus exhibits a structured and predictable form of nonlinear behavior: the parameter-driven partitioning of the voltage axis into distinct flux response regimes.

Practically speaking, our findings offer concrete criteria that could be used to interpret or design experiments involving ionic flows through channels or nanopores. The critical potential $V_{k}^{a}$ for each ion, being directly related to that ion’s concentration gradient across the membrane, can be viewed as an approximate voltage scale at which the influence of fixed charge on that ion’s current reverses. In experimental terms, if one were to introduce a small amount of fixed charge into a channel (for example, via site-directed mutagenesis adding a charged residue, or by doping a nanopore with charged functional groups), the theory predicts that the resulting change in the ion’s I-V curve will switch from a positive effect to a negative effect around the voltage $V=V_{k}^{a}$. Because $V_{k}^{a}$ depends only on readily measurable quantities, one could estimate this critical voltage beforehand and thus anticipate the qualitative outcome of such modifications. This has implications for ion channel engineering: to enhance the conductance of a particular ion species in a certain operating voltage range, one would ensure the applied voltages stay on the side of $V_{k}^{a}$ where the fixed charge’s effect is beneficial; to suppress unwanted ion flux, one might operate on the opposite side of that threshold. Meanwhile, the additional critical potentials $V^{b}$ and $V^{c}$, which arise from multi-ion interactions, suggest that channels with multiple permeant ion species can exhibit more than one transition in their response. Although $V^{b}$ and $V^{c}$ are defined through model-specific parameter combinations and are not as straightforward to infer from basic experimental data, their existence implies that features such as inflection points or slope changes in measured I–V curves could be tied to the underlying thresholds predicted by the model. In practice, observing a change in how a small fixed charge perturbs the current-voltage relationship (for instance, a fixed charge that helps current at low voltages but hinders it at high voltages) would be strong evidence of the voltage-partitioning effect quantified by $V_{k}^{a},V^{b},$ and $V^{c}$.

It is worth emphasizing that all these conclusions hold under the relaxed electroneutrality boundary conditions employed in this study, which allow for slight charge imbalance at the channel boundaries. The persistence of clearly defined critical potentials in this generalized setting speaks to their fundamental nature in governing ionic flows. In fact, relaxing the electroneutrality constraint makes our results more broadly applicable, as real biological and synthetic channels may not always maintain perfect electroneutrality in their boundary reservoirs. The ability to pinpoint critical voltages for flux enhancement or suppression without requiring strict electroneutrality suggests that the phenomena described are robust and not an artifact of overly idealized conditions. In summary, the identification of $V_{k}^{a},V^{b}$, and $V^{c}$ enriches the theoretical framework for ionic transport by delineating precise conditions for qualitative changes in flow. These critical potentials deepen our understanding of how permanent charge and transmembrane potential conspire to regulate ion movement, and they bridge the gap between mathematical analysis and experimental observables by providing testable predictions for multi-cation PNP systems. The insights gained here lay a foundation for future investigations into controlling ionic currents, either by tuning channel fixed charges or by modulating operating voltages, in technologies and biological scenarios where multiple ion species compete and interact under non-ideal boundary conditions.

## 5. Conclusions

In summary, we have developed a one-dimensional Poisson–Nernst–Planck framework for ionic flow through a channel with multiple cation species under relaxed electroneutrality boundary conditions. By allowing the reservoir boundary conditions to be nearly−but not exactly−electroneutral, our model captures the subtle boundary layer effects that are typically lost under the conventional assumption of strict electroneutrality. The mathematical analysis employs a two-step perturbation approach: first, a singular perturbation (geometric singular perturbation) method is used to derive explicit leading-order solutions for the ionic fluxes, accounting for the sharp boundary-layer structure; second, a regular perturbation expansion is performed around the near-neutral state (with small deviations in reservoir charge balance and a small permanent charge). This asymptotic approach yields analytical expressions for the individual ion fluxes and provides a rigorous handle on how small permanent charges and slight boundary concentration imbalances affect transport.

A key outcome of this study is the identification of several critical potential values—denoted $V_{k}^{a}$ (for each cation species *k*), $V^{b}$, and $V^{c}$—that fundamentally govern the qualitative behavior of the ionic fluxes. These critical potentials partition the applied voltage axis into distinct regimes, in each of which the influence of a fixed (permanent) charge on ion flow is qualitatively different. In particular, the characteristic potential $V_{k}^{a}$ (determined by the valence of ion *k* and the ratio of its boundary concentrations) emerges as a threshold delineating whether the presence of a small positive permanent charge enhances or reduces the flux of cation *k*. The relative positioning of $V_{k}^{a}$ with respect to the global threshold $V^{b}$ determines the sign of the fixed charge’s effect on that cation’s current across various voltage ranges. Likewise, for the anion, the interplay between its own characteristic potential $V_{3}^{a}$ and another critical value $V^{c}$ dictates how the permanent charge modulates the anionic flux. In essence, $V^{b}$ and $V^{c}$ serve as additional voltage landmarks (arising from the coupling of multiple ion species and channel parameters) that, together with $V_{k}^{a}$, mark transitions in flow behavior. The explicit formulas derived for these potentials not only underscore their theoretical importance but also make them directly computable from experimental parameters−for example, $V_{k}^{a}$ can be readily calculated from known ionic valences and concentration ratios, providing a tangible link between our theory and measurable quantities in ion channel experiments.

This analysis yields several theoretical insights into how a small permanent charge interacts with an applied electric potential in a multi-ion channel setting. Notably, we find that a slight permanent charge does not simply exert a uniform bias on ionic transport; instead, its effect is highly voltage-dependent and can even reverse sign depending on the regime. For voltages below or above certain critical values, the presence of a small positive fixed charge may facilitate (augment) the flux of a given ion, whereas in the intermediate range between critical potentials it may inhibit (diminish) that flux. This nuanced behavior highlights a form of built-in gating or regulatory mechanism: the channel’s permanent charge can selectively boost or suppress currents in different voltage ranges. Such findings deepen our understanding of ion channel selectivity and rectification, revealing that even a very small fixed charge, when combined with near-neutral boundary conditions, can induce complex nonlinear responses in the current-voltage relationship.

Beyond the mathematical characterization, our results carry practical implications for modeling ion channels and interpreting their transport behavior. The ability to pinpoint critical potentials means that one can predict under what conditions a channel’s fixed charge will have pronounced effects on ionic conductance. For instance, knowing the values of $V_{k}^{a}$ for the cation species permeating a channel allows experimentalists to anticipate the voltage at which each ion’s current may reverse or experience maximal modulation due to the channel’s fixed charges. More generally, the framework presented here provides a more realistic baseline for ion channel modeling by incorporating the influences of boundary layer charge accumulation and small permanent charges. This can improve the interpretation of experimental I-V data: features such as current rectification, plateaus, or sudden changes in slope can be traced to the analytically identified critical voltages. In summary, by combining singular perturbation analysis with a relaxed electroneutrality model, we have illuminated how subtle factors−slight deviations from reservoir electroneutrality and minuscule fixed charges−can lead to qualitative changes in ionic fluxes. These findings enrich the theoretical foundation of ionic flow in channels and offer clear, testable predictions, thereby bridging mathematical insight with the physiological understanding of ion transport systems.

To end this section, we demonstrate that while our model is relatively simple and may not fully capture the complexity of crowded ionic mixtures, this deliberate simplification was necessary to achieve our primary objective: to analyze the concrete effects of small permanent charges under relaxed neutral conditions in terms of some critical potentials. This approach is justified in the context of semiconductor problems and synthetic channels and provides a valuable first step toward more realistic models. The analytical insights gained from this simpler setting, particularly the explicit expressions for ionic fluxes, are foundational for further research and the development of more complex models.

## Data Availability

The original contributions presented in this study are included in the article. Further inquiries can be directed to the corresponding authors.

## Abbreviations

The following abbreviations are used in this manuscript:

PNP	Poisson–Nernst–Planck
